# Nutrition, Safety and Storage of Seafoods

**DOI:** 10.3390/foods15122058

**Published:** 2026-06-07

**Authors:** Zhe Xu, Deyang Li

**Affiliations:** 1 Key Laboratory of Biotechnology and Bioresources Utilization, College of Life Sciences, Dalian Minzu University, Ministry of Education, Dalian 116600, China; xuzhe@dlnu.edu.cn; 2 State Key Laboratory of Marine Food Processing & Safety Control, National Engineering Research Center of Seafood, Collaborative Innovation Center of Seafood Deep Processing, School of Food Science and Technology, Dalian Polytechnic University, Dalian 116034, China

Marine resources are abundant and are often referred to as humanity’s second granary, playing a key role in optimizing food production through their development. As a core component of these resources, current processes for the processing, utilization, and storage of seafood often lead to nutrient loss and wastage of bioactive substances. This Research Topic aims to bring together the latest findings on the processing and utilization of marine food resources. It seeks to minimize nutrient loss and sensory deterioration during processing and storage through advanced technologies, efficiently extract bioactive substances to enhance the utilization of seafood by-products, and develop strategies for optimizing deep processing and utilization. Furthermore, investigations into changes in functional quality and nutrition will provide a theoretical basis for the further development of functional foods based on marine food resources.

With the growth of the global population, the demand for food production will increase. In this context, the development of marine food resources will become a significant part of food production [1]. However, limitations in production processes and technologies can easily lead to food quality and safety issues in seafood by-product foods [2,3]. On the other hand, during the manufacturing, storage, transportation, and distribution of seafood products, traditional processing methods can lead to quality deterioration, failing to preserve nutritional properties and sensory qualities, thereby reducing their economic value [4,5,6,7]. The limitations in production processes and technologies highlights the urgent need for innovative preservation technologies. Although seafood is nutritious, there is a need to innovate new products to consolidate its use as a functional ingredient, with pretreatment playing a key role in preserving nutrient [8,9].

This Research Topic comprises 9 published papers. The manuscripts indicate that the processing technologies and techniques for marine food resources constitute a key research area.

Among the articles in this Research Topic, topics covered include the development and utilization of marine food resources, extraction and functional evaluation of food bioactive components, quality control and safety evaluation during food processing, and methods for the storage and preservation of marine food products. Improving drying techniques for brown seaweed, such as vacuum, infrared, or microwave-assisted drying, was shown to enhance the energy efficiency and chemical stability of bioactive compounds, improving preservation outcomes [1]. Fish sauce, a traditional fermented condiment in Southeast and East Asia, has quality and safety significantly influenced by factors such as raw materials, fermentation process, and origin. Products from different origins showed significant differences in chemical composition. For example, Zarei et al. [10] reported the average iodine content of fish sauce from five different production areas in southern Iran was 2662 mg/kg. Jiang et al. [11] reported the concentrations of histamine (Him), putrescine (Put), cadaverine (Cad) and tyramine (Tyr), the major biogenic amines (BAs) detected in fish sauce products from three Chinese provinces, were all above 100 mg/kg. The study quantified the content standards of key substances, emphasizing the importance of monitoring key contents in fish sauce and accurate product labeling to comply with food safety regulations, which helps strengthen food supervision, improve quality control, and promote safer production and consumption of fish sauce [2]. In dried laver production, background microorganisms from laver and seawater proliferate along the production line with the release of laver nutrients. Continuous processing without adequate routine cleaning and disinfection, compounded by weather conditions, further promoted microbial proliferation and cross-contamination, leading to high microbial loads and unique microbial communities on dried laver products and food contact surfaces [3]. Applying ultra-high pressure (UHP) treatment to fish balls proved an efficient method for improving product quality. After 0–5 freeze-thaw (F-T) cycles, the physicochemical and gelation properties, as well as color, of UHP-treated fish balls were superior to those made by the traditional two-step heating process. UHP treatment achieved uniform ice crystal distribution, slowed ice crystal growth, preserved muscle tissue integrity, and enhanced the textural properties of the fish balls through a more regular arrangement of myofibrils [4].The addition of natural extracts, such as fish skin antifreeze peptides and rosemary extract, can effectively inhibit lipid oxidation formation and microbial growth, maintain physicochemical and sensory properties, and significantly extend the storage time [5]. The synergistic effect of UHP and egg white protein (EWP) addressed issues of weak gel strength and poor water-holding capacity in low-salt shrimp surimi caused by salt reduction, ensuring product quality while reducing salt content by 40%, aligning with the salt reduction needs of healthy diets [6]. When refrigerating Trachurus trachurus fillets, incorporating *Gelidium* sp. into a gelatin packaging system produced a preservative effect; samples with algal powder exhibited significantly lower microbial development. Chemical indicators related to microbial development (pH and trimethylamine) were also significantly reduced, peroxide retention was significantly higher, and the formation of secondary oxidation products was significantly reduced. The algae flour group showed significantly lower lipid hydrolysis [7]. Cellulose saccharification of brown seaweed (*Lessonia spicata*) for developing a functional fermented beverage optimized the saccharification process using response surface methodology to obtain optimal conditions, finding that enzymatic saccharification significantly enhanced the fermentation performance and functional properties of the beverage [8]. The optimization of extraction of substances such as bound polyphenols and polysaccharide extracts from algae showed high inhibitory activity against α-glucosidase and high free radical scavenging activity in antioxidant assays. These results provide valuable basic data for the application of macroalgae residues in the marine bio-industry and reveal their potential hypoglycemic capacity [9]. However, the aforementioned reports still focus on the development of marine food resources and the identification of existing processing problems. There remains a need for better elucidation of the underlying mechanisms and further experimentation to optimize conditions. Therefore, further research is necessary on the in vivo bioavailability, sensory acceptance, and gut microbiota modulation of bioactive compounds from marine resources to validate the potential health benefits and commercial viability of products. Additionally, there is a need to enhance the quality and safety inspection of seafood products and establish unified testing standards to comply with food safety regulations. This will play an important role in strengthening food supervision, improving quality control, and promoting the safer production and consumption of seafood products.

In summary, this Research Topic contributes to a better understanding among researchers regarding the development and utilization of marine food resources. However, the in vivo bioavailability, sensory evaluation, and safety concerns of these products require further determination, and the mechanisms of action of functional products also need further clarification.

## Data Availability

Not applicable.
